# Comment on “Naples Prognostic Score and Clinical Outcomes After PCI for Acute Coronary Syndrome: A Systematic Review and Meta‐Analysis”

**DOI:** 10.1002/clc.70270

**Published:** 2026-02-01

**Authors:** Bin Cao, Rengyun Xiang

**Affiliations:** ^1^ Department of Cardiology The Central Hospital of Yongzhou Yongzhou China; ^2^ Department of Cardiology Yongzhou Hospital Affiliated to University of South China Yongzhou China; ^3^ The First Affiliated Hospital of Jishou University Jishou China

Dear Editor,

We read with interest the meta‐analysis by Özen et al. on the Naples Prognostic Score (NPS) in ACS patients undergoing PCI [1]. The study confirms that a higher NPS is associated with increased mortality, no‐reflow, and lower LVEF, contributing to the field of biomarker‐based risk stratification. We wish to highlight several limitations that merit further discussion.

First, the study overlooks the methodological limitation of dichotomizing continuous biomarkers. The NPS components (e.g., albumin, NLR) are continuous and may have non‐linear relationships with outcomes. Forcing them into binary categories loses gradient risk information and masks component interactions. For instance, different combinations of albumin and NLR levels may yield the same NPS but carry distinct risks. This oversimplification likely contributes to the high heterogeneity observed, particularly for LVEF. Birdal et al. showed that while the high‐NPS group had lower LVEF (45.2 ± 8.7% vs. 50.1 ± 8.2%, *p* < 0.001), the impact of individual components varied significantly—a nuance obscured by dichotomization [2]. Thus, future studies should treat biomarkers as continuous variables and use advanced models (e.g., splines, machine learning) to capture non‐linear relationships and enable personalized risk stratification.

Second, the study neglects that the NPS's simple summation of nutrition (albumin, cholesterol) and inflammation (NLR, LMR) markers may blur distinct pathophysiologies (“nutritional depletion” vs. “inflammatory storm”). Keskin et al. identified two high‐NPS subtypes in NSTEMI patients: a “low‐albumin, low‐inflammation” group with higher heart failure/infection rates, and a “normal‐albumin, high‐inflammation” group with higher reinfarction rates [3]. Targeted interventions yielded subtype‐specific benefits (nutrition support reduced heart failure risk by 41% in the former; anti‐inflammatory therapy reduced reinfarction by 53% in the latter). These critical differences are masked by the total NPS. Future work should use methods like cluster analysis to identify such phenotypes and test targeted therapies, transforming the NPS from a “black‐box” score into a actionable phenotyping tool.

Third, the study underemphasizes that current evidence is largely observational, limiting causal inference. It remains unclear whether a high NPS directly contributes to poor outcomes or merely reflects overall disease severity. Genc et al. found that after adjusting for disease severity markers, the independent association between NPS and prolonged hospitalization was attenuated (p‐value increased from < 0.01 to 0.08) [4], suggesting NPS may be a severity marker rather than an independent driver. Therefore, the next step should be prospective validation and randomized trials testing whether NPS‐guided interventions improve hard endpoints, elevating it from a predictor to an intervention target.

Finally, the study does not define clinical action thresholds. While confirming “high NPS = high risk,” it does not specify at what score to escalate care or which interventions to start. This gap limits its integration into clinical pathways. Future research should design trials (e.g., randomizing patients with NPS ≥ 3 to intensified vs. routine care) to determine if NPS‐stratified management improves outcomes, bridging the gap between prognostic association and clinical utility.

In summary, while Özen et al. systematically confirm the NPS's prognostic value, deeper questions remain. The focus should now shift from validating associations to elucidating mechanisms, enhancing evidence through prospective/interventional studies, and verifying clinical utility by defining actionable thresholds. This translation is essential for the NPS to evolve from a prognostic marker into a tool for guiding individualized ACS management.

## Author Contributions

All the authors contributed to the study conception and design. The first draft of the manuscript was written by Bin Cao, and all the authors commented on previous versions of the manuscript. All the authors read and approved the final manuscript.

## Funding

The authors received no specific funding for this work.

## Conflicts of Interest

The authors declare no conflicts of interest.

## Data Availability

This article does not generate any original data. All data supporting the findings of this study are included within the article and are sourced from previously published literature, which has been appropriately cited.
